# Synthesis of Hydroxymethylenebisphosphonic Acid Derivatives in Different Solvents

**DOI:** 10.3390/molecules21081046

**Published:** 2016-08-11

**Authors:** Dávid Illés Nagy, Alajos Grün, Sándor Garadnay, István Greiner, György Keglevich

**Affiliations:** 1 Department of Organic Chemistry and Technology, Budapest University of Technology and Economics, 1521 Budapest, Hungary; duwa24@gmail.com (D.I.N.); agrun@mail.bme.hu (A.G.); 2 Gedeon Richter Plc., 1475 Budapest, Hungary; garadnay@richter.hu (S.G.); i.greiner@richter.hu (I.G.)

**Keywords:** hydroxymethylenebisphosphonic acid (dronic acid) derivatives, solvent, P-reagents, synthesis

## Abstract

The syntheses of hydroxymethylenebisphosphonic acid derivatives (dronic acid derivatives) starting from the corresponding substituted acetic acids and P-reagents, mainly phosphorus trichloride and phosphorous acid are surveyed according to the solvents applied. The nature of the solvent is a critical point due to the heterogeneity of the reaction mixtures. This review sheds light on the optimum choice and ratio of the P-reactants, and on the optimum conditions.

## 1. Introduction

The hydroxymethylenebisphosphonic acid derivatives (dronic acid derivatives) form an important group within organophosphorus pharmaceutics. It is known that the two phosphonic functions are capable of forming complex with calcium ions, hence, the resorption of these ions is prevented. Bisphosphonic derivatives are used in the treatment of osteoporosis, Paget disease and tumor-induced hypercalcaemia, but they also show direct antitumor and antiparasitic activity [1,2,3,4,5,6].

Although the synthesis of dronic acid derivatives is widely discussed in the literature, it can be considered a sort of “black box”. They were prepared in many solvents, but the optimum circumstances, and the molar ratios of the P-reagents were not explored. The role of the reagents and the reaction mechanism were not clarified. In most cases, the purity of the products was not reported, or when crude products were prepared, unrealistically high, and hence misleading yields were claimed. We wish to summarize the preparation of the most important hydroxymethylenebisphosphonic acid derivatives according to solvents, hoping to make the syntheses more transparent. The most important therapeutic agents discussed in this review are listed in Table 1.

**Scheme 1:**
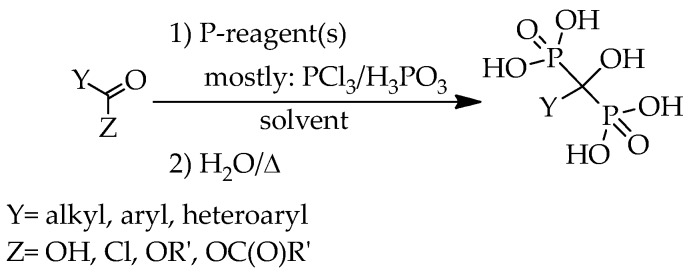
General scheme for the preparation of hydroxymethylenebisphosphonic acids.

According to the general scheme for the preparation of hydroxymethylenebisphosphonic acid derivatives, the corresponding carboxylic acid, or its chloride, ester and anhydride derivative was used as the starting material, and reacted with phosphorus trichloride and/or phosphorous acid, phosphoric acid or phosphoryl chloride in a wide variety of solvents, for example methanesulfonic acid (MSA), sulfolane, chlorobenzene, toluene, xylene, *n*-octane, methyl cyclohexane, 1,4-dioxane, acetonitrile, phenol and its derivatives, alkyl carbonates, phosphates, silicon or sunflower oil, ionic liquids, and also in the absence of any solvent (Scheme 1).

In many publications, water was applied as the solvent, and phosphorus trichloride as the reagent, which is absolutely pointless, as the water reacts fast with the P-reagent to provide phosphorous acid [7,8,9,10,11]. The concept of generating phosphorous acid “in situ” may be questioned, as this approach is expensive, pollutes the environment, and rather dangerous on the industrial scale. We did not deal with this not too practical method.

## 2. Reactions in Methanesulfonic Acid (MSA)

**Scheme 2:**
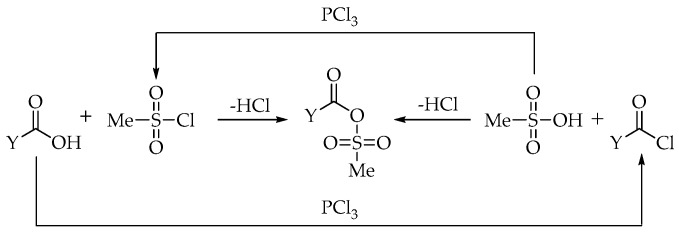
Possible formation of carboxylic acid-MSA mixed anhydride as an intermediate.

MSA may be considered the best solvent during the synthesis of dronic acid derivatives, as it helps to overcome the problems of heterogeneity during the reaction. However, the MSA is likely to participate in the reactions themselves. It can activate the carboxylic acid to form a carboxylic acid-MSA mixed anhydride, which may be formed in two ways, as shown in Scheme 2 [12].

**Scheme 3:**
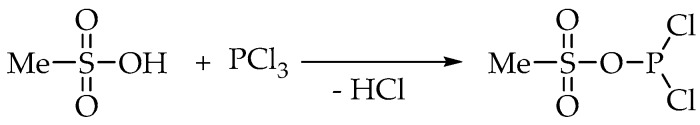
Activation of phosphorus trichloride by reaction with MSA.

MSA may also react with phosphorus trichloride to provide a more reactive P-species (Scheme 3).

**Scheme 4:**
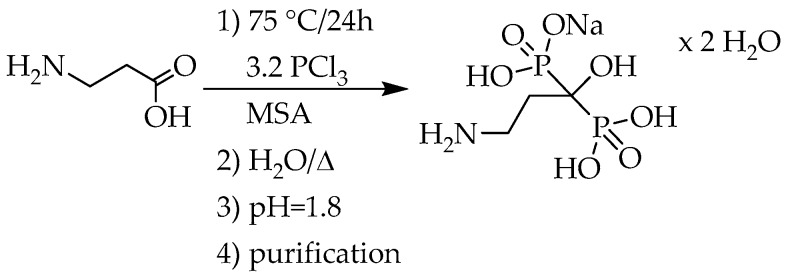
Preparation of pamidronate in MSA by an optimized method [16].

In all syntheses of pamidronate described, β-alanine was applied as the starting material in MSA (Scheme 1, Y = −CH_2_CH_2_NH_2_, Z = −OH, solvent: MSA). Szűcs, Vepsäläinen and his co-workers used 2 equivalents of phosphorus trichloride and 1 equivalent of phosphoric acid as the P-reagents (Table 2/Entries 1 and 2) [13,14]. Shinkai et al. applied phosphorus trichloride and phosphorous acid in ratio of 2.1:1 (Table 2/Entry 3) [15]. In the lack of purification, no purities were provided. The best result was achieved by Keglevich et al. who proved that the phosphorous acid did not participate in the reaction under discussion due its low nucleophilicity. For this, only 3.2 equivalents of phosphorus trichloride were measured in. The yield of pamidronic acid monosodium salt trihydrate was 57%, and the purity was 99% (Scheme 4) (Table 2/Entry 4) [16].

The preparations of alendronic acid derivatives were also studied in details using MSA. In most cases γ-aminobutyric acid (GABA) was treated with the P-reagents (Table 3/Entries 1–9) [14,15,17,18,19,20,21,22,23]. In one instance, 2-pyrrolidone was the starting material (Table 3/Entry 10) [24].

**Scheme 5:**
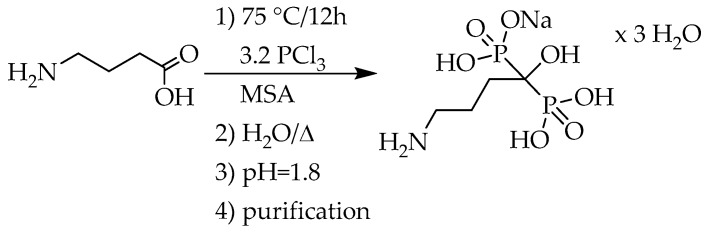
Preparation of alendronate in MSA by an optimized method [21].

The majority of the researchers applied phosphorus trichloride and phosphorous acid as reagents in a ratio of ca. 2:1 (Scheme 1, Y = −CH_2_(CH_2_)_2_NH_2_, Z = −OH, solvent: MSA) (Table 3/Entries 1–3, 5 and 6) [15,18,19,20,23]. Yields around 80%–90% were claimed. As in the case of pamidronate, Vepsäläinen and his co-workers used 2 equivalents of phosphorus trichloride and 1 equivalent of phosphoric acid (Table 3/Entry 4) [14]. The best reproducible results were reported by Keglevich et al., furnishing alendronate in a yield of 58% (Scheme 5) (Table 3/Entry 7) [21]. It was clarified that phosphorous acid is unnecessary, when phosphorus trichloride is the P-reagent and MSA is the solvent.

Typically, the yields were higher than 80% for the crude product (Table 3/Entries 1–6 and 10) [14,15,18,19,20,23,24]. In only two cases were the purities reported, however, the values of 99.7% seem to be excessive (Table 3/Entries 2 and 3) [15,23]. Our experience is that a crude product may never be pure, and such high values are not realistic.

In a special combination, phosphoryl chloride and phosphorous acid were used in a proportion of 2:3 or 3:3 in MSA (Table 3/Entry 8) [22]. In one case, 2 equivalents of phosphorous acid and 1 equivalent of phosphorus pentoxide were the P-reactants, and the target dronate was obtained in a yield of 48% (Table 3/Entry 9) [17]. Using 2-pyrrolidone as the starting material together with 3.4 equivalents of phosphorus trichloride, the crude alendronate was isolated in a yield of 81% (Table 3/Entry 10) [24].

**Scheme 6:**
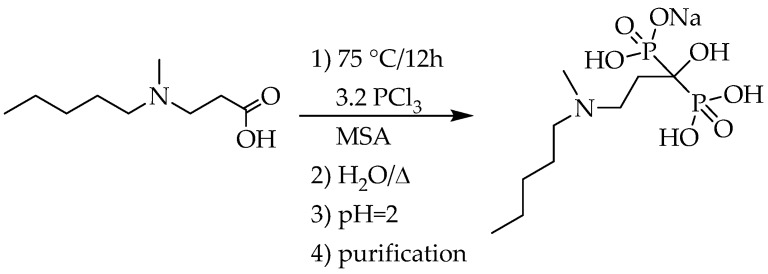
Preparation of ibandronate in MSA by an optimized method [21].

The synthesis of ibandronate in MSA was discussed in only a few publications. In these preparations, *N*-Methyl-*N*-pentyl-β-alanine (MPA) was treated with phosphorus trichloride and phosphorous acid under different conditions (Scheme 1, Y = −CH_2_CH_2_N(Me)(Pen), Z = −OH, solvent: MSA). Soni et al. applied 5 equivalents of phosphorus trichloride together with 2 equivalents of phosphorous acid. According to their description, pure alendronate was isolated in a yield of 86% (Table 4/Entry 1) [25]. The use of phosphorus trichloride is completely unnecessary. Applying 2.5 equivalents of phosphorus trichloride, and the same amount of phosphorous acid in the mixture of MSA and chlorobenzene, the yield was 60% (Table 4/Entry 2) [26]. The optimum case was, when 3.2 equivalents of phosphorus trichloride were used. The realistic and reproducible yield was 46% (Scheme 6) (Table 4/Entry 3) [21].

For risedronic acid derivatives, yields of 38%–74% were reported. In the basic syntheses, 3-pyridylacetic acid (PAA) was reacted with phosphorus trichloride and phosphorous acid in MSA (Scheme 1, Y = 3-pyridylmethylene-, Z = −OH, solvent: MSA).

**Scheme 7:**
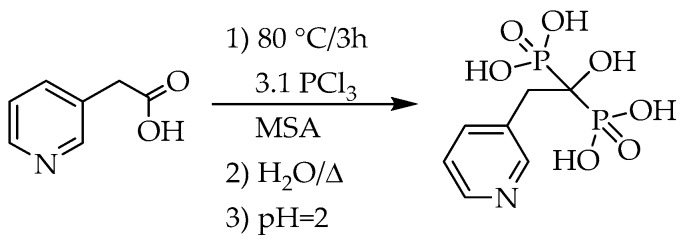
Preparation of risedronic acid in MSA by an optimized method [28,29].

A yield of 73% was obtained, when 3-3 equivalents of phosphorus trichloride and phosphorous acid were used in the mixture of MSA and diethyl carbonate (Table 5/Entry 1) [27]. Another combination, a 2.1:1 ratio of the P-reagents led to the dronic acid in a yield of 38%. No purity was reported (Table 5/Entry 2) [15]. When the mixture of 3 equivalents of phosphorous acid and 2 equivalents of phosphoryl chloride, or 2 equivalents of phosphorous acid and 1 equivalent of phosphorus pentoxide were reacted with the corresponding carboxylic acid, yields of ca. 55% were reported (Table 5/Entries 3 and 4) [17,22]. The application of phosphorus trichloride (3.1 equivalents) alone led to the highest yield (74%), and the unnecessity of phosphorous acid was again proved (Scheme 7) (Table 5/Entry 5) [28,29].

**Scheme 8:**
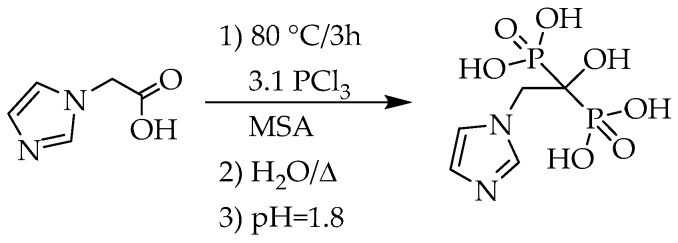
Preparation of zoledronic acid in MSA by an optimized method [28,29].

During the preparation of zoledronic acid in MSA, imidazol-1-yl-acetic acid (IAA) was treated with only phosphorus trichloride, or with the mixture of this reagent and phosphorous acid (Scheme 1, Y = 1-imidazolylmethylene-, Z = −OH, solvent: MSA). Kieczykowski and co-workers applied phosphorus trichloride and phosphorous acid in a quantity of 2.1:1 equivalents. The yield of the target dronic acid was 31% (Table 6/Entry 1) [15]. Using phosphorus trichloride as the reactant in amounts of 3.1 or 3.2, yields of 53% and 46%, respectively, were reported by the Keglevich group in comparable purity (Scheme 8) (Table 6/Entries 2 and 3) [28,29].

In overall, it can be said that the lower yields of the Keglevich group are better, than the higher ones published for the crude products neglecting the purity. The reliable yields were obtained after purifying the products and analyzing the purity by potentiometric titration.

## 3. Synthesis of Dronic Acid Derivatives in Chlorobenzene

**Scheme 9:**
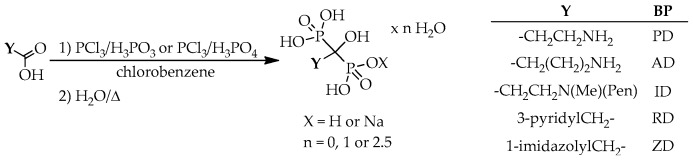
General scheme for the preparation of dronic acid derivatives in chlorobenzene.

The second frequently used solvent is chlorobenzene. Chlorobenzene has a few advantages in comparison with MSA. It has a lower viscosity, can be removed by distillation, and can be reused. The real problem with MSA is that during the pH adjustment sodium methanesulfonate may be formed from MSA, which is difficult to remove. In the procedures reported, the corresponding carboxylic acids were reacted with phosphorus trichloride and phosphorous acid/phosphoric acid. None of the reactions were performed with solely phosphorus trichloride, as, in this case, it is unreactive without another P-containing partner (Scheme 9). In most reports no data on the purity of dronic acid derivatives was provided, for this, the yields are not reliable.

Reacting β-alanine or GABA with 1.5 equivalents of phosphorus trichloride and 1.5 equivalents of phosphorous acid at 100 °C for 3 h, the yields were in the range of 45%–59% (Table 7/Entries 1 and 3–5) [30,31,32]. Starting from β-alanine or GABA, and measuring in phosphorus trichloride and phosphoric acid in molar equivalents of 2.4:1.5 or 2:3, respectively, the pamidronic acid or alendronic acid was obtained in a yield of 53% and 18%, respectively (Table 7/Entries 2 and 6) [33,34].

In two variations for the syntheses of ibandronate, a yield of 60% was reached. Phosphorus trichloride and phosphorous acid were applied in a 1.5:1.5 or 2.5:2 molar equivalent quantity (in a mixture of MSA and chlorobenzene) (Table 8/Entries 1 and 2) [26,35]. Using phosphorus trichloride and phosphoric acid in amounts of 3:3 and 2:3, the yield was 37% and 6%, respectively (Table 8/Entries 3 and 4) [34,36]. In one instance, the proportion of phosphorous acid was not provided, and a peripheral result (a yield of 3%) was reported (Table 8/Entry 5) [26].

The preparation of risedronic acid derivatives in chlorobenzene as the solvent was in all reactions implemented using phosphorus trichloride and phosphorous acid. An unrealistically high yield of 96% was claimed for risedronic acid using 3.5 equivalents of phosphorus trichloride, and the same amounts of phosphorous acid without specifying the purity (Table 9/Entry 1) [37]. Applying the P-reagents in a quantity of 1.5 equivalents, the yield was 68% (Table 9/Entry 2) [35]. The most reliable result was described by Wadhwa, but the reaction time was not given. The reactants were used in amount of 2.4 equivalents, and the risedronic acid was obtained in a yield of 58% with a purity of 84% (Table 9/Entry 3) [38]. Applying phosphorus trichloride and phosphorous acid in a molar ratio of 3:3.5, the dronate was isolated in a yield of 52%. However, no temperature was reported. A similar yield (52%) was described, when 2 equivalents of phosphorus trichloride was used with phosphorous acid. However, the quantity of the latter species was not provided (Table 9/Entries 4 and 5) [39,40].

For the synthesis of zoledronic acid in chlorobenzene, the purity remains unclear in all cases, therefore, the yields are not reliable at all. Carrying out the synthesis with 3.7 equivalents of phosphorus trichloride and with the same amount of phosphorous acid, quantitative yield was claimed that is entirely unrealistic (Table 10/Entry 1) [41]. In other instances, phosphorus trichloride and phosphoric acid were applied in different, such as 4.7:2.9, 2:3 and 3:1.9 ratios to lead to yields of 79%, 67% and 41%, respectively (Table 10/Entries 2–4) [34,42,43].

The need for the joint application of phosphorus trichloride and phosphorous acid will be explained in the next sub-chapter.

## 4. Methods for the Preparation of Dronic Acid Derivatives in Sulfolane

**Scheme 10:**
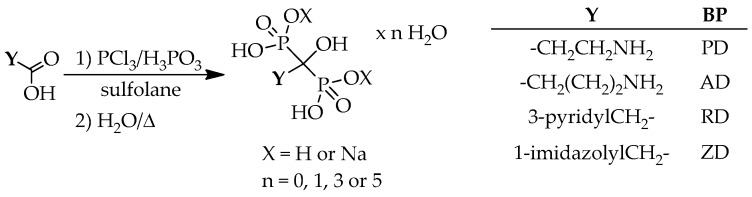
General scheme for the preparation of dronic acid derivatives in sulfolane.

Sulfolane is also a preferred solvent in the preparation of hydroxymethylenebisphosphonic acid derivatives, but less data were published. In all such cases, phosphorus trichloride and phosphorous acid were the P-reagents (Scheme 10).

Approximately in half of the cases, the purities were not described, therefore the data may be misleading. The synthesis of a part of the dronic acid derivatives was attempted also under MW conditions. The synthesis of ibandronic acid derivatives was not attempted in sulfolane.

Applying phosphorus trichloride and phosphorous acid in a molar ratio of 3:3, under conventional and MW-assisted conditions at 65 °C for 3.5 h and 3 min, respectively, yields of 72% and 64%, respectively, were reported for crude pamidronic acid derivatives (Table 11/Entries 1 and 2) [44]. (The forms of product were not provided.) Alendronate was obtained under similar conditions in yields of 38% and 41%, respectively (the forms of product were not provided) (Table 11/Entries 7 and 8) [44]. It can be seen that MW irradiation was useful in respect of reaction time in these reactions. Keglevich et al. used 2-2 equivalents of the P-reagents in reaction with β-alanine to afford pure pamidronic acid in a yield of 63% (Table 11/Entry 3) [16]. When phosphorus trichloride and phosphorous acid were used in a ratio of 3.4:1.5, pure sodium pamidronate was obtained in a lower yield (Table 11/Entry 4) [45]. Applying phosphorus trichloride and phosphorous acid in ratios of 3.4:1.5 and 2.5:3.5, the yield of sodium alendronate was 69% (crude product), and 55% (pure dronate), respectively (Table 11/Entries 5 and 6) [45,46].

McKenna and his co-workers investigated the synthesis of risedronic and zoledronic acid derivatives (the forms of product were not provided) also under MW conditions, using 3 equivalents of both the phosphorus trichloride and phosphorous acid at 65 °C for 3 min. The dronic acid derivatives, risedronic acid and zoledronic acid derivatives were obtained in yields of 74% and 70%, respectively (Table 12/Entries 1 and 3) [44]. In case of conventional heating, the yield of zoledronic acid derivative was 67% (Table 12/Entry 4) [44]. The reaction of 1 equivalent of PAA or IAA with 3.4 equivalents of phosphorus trichloride and 1.5 equivalents of phosphorous acid on conventional heating at around 65 °C provided risedronate and zoledronic acid in a yield of 54% and 71%, respectively (Table 12/Entries 2 and 5) [45]. No attempts were made to prepare pure zoledronic acid derivatives.

**Scheme 11:**
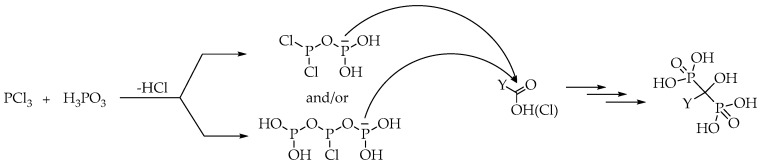
Formation of the reactive P-species by the condensation of phosphorus trichloride and phosphorous acid and their reaction with the substituted acetic acids.

It was shown in Chapter 2 that using MSA as the solvent, there was no need to use phosphorous acid together with phosphorus trichloride (Table 2, Table 3, Table 4, Table 5 and Table 6) [16,21,28,29]. However, using a solvent other than MSA, it is necessary to apply phosphorus trichloride and phosphorous acid jointly. A logical explanation may be that, before phosphorus trichloride would react with the carboxylic acid or its derivatives, it reacts with 1 or 2 equivalents of phosphorous acid resulting in the formation of activated P-species, such as (HO)_2_P-O-PCl_2_ and/or (HO)_2_P-O-PCl-O-P(OH)_2_ (Scheme 11). In the next step, the reactive intermediates formed may react with the substituted acetic acid, providing the target dronic acid derivative after several steps [16].

## 5. Synthesis of Dronic Acid Derivatives in Toluene

**Scheme 12:**
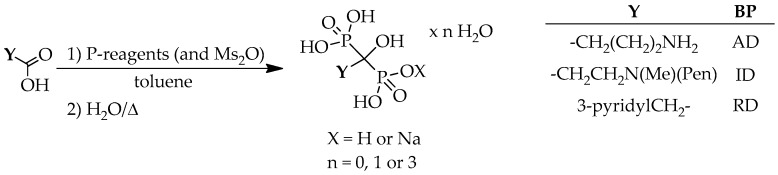
General scheme for the preparation of dronic acid derivatives in toluene.

The preparations of alendronic acid derivatives, ibandronate and risedronic acid have also been investigated in toluene (Scheme 12).

In one approach towards alendronic acid, a mixture of toluene and PEG 400 was used as the solvent, and 1.6 equivalents of phosphorus trichloride along with 1 equivalent of phosphorous acid as the P-reagents. The yield was 56% for crude alendronic acid (Table 13/Entry 1) [47]. Using phosphorus trichloride and methanesulfonic anhydride (Ms_2_O) in a molar ratio of 3:3 in toluene, a yield of 66% was reported (Table 13/Entry 2) [48]. The application of 3.7 equivalents of phosphorus trichloride and 4.2 equivalents of phosphorous acid led to a moderate yield (44%) of sodium ibandronate (Table 13/Entry 3) [49].

Regarding the synthesis of risedronic acid, in most instances unusual reagents for example, propylphosphonic anhydride (T3P), Ms_2_O or PCl_5_ were used together with phosphorous acid, and purities of >90% were reported according to HPLC (Table 14/Entries 1–6). Unrealistically high yields of 90% and 81% were reported, when 3.1 equivalents phosphorous acid were reacted with 2.5 equivalents of T3P or 3.1 equivalents of phosphoryl chloride (Table 14/Entries 1 and 2) [50]. In similar reactions, with ca. 3 equivalents of reactants (phosphorous acid and Ms_2_O) a lower yield of 34% was obtained (Table 14/Entry 3) [50]. Other interesting combinations, such as a 3.1:2:1 ratio of H_3_PO_3_:Ms_2_O:POCl_3_, or a 3.1:1:1 ratio of H_3_PO_3_:Ms_2_O:PCl_5_ afforded the risedronic acid in yields of ca. 60% (Table 14/Entries 4 and 5) [50]. The application of 3.2 equivalents of phosphorus trichloride alone led to the lowest yield (15%) (Table 14/Entry 6) [12]. It seems to have been confirmed that phosphorus trichloride alone is not enough if not MSA is the solvent. In the cases discussed, it was necessary to apply an activating agent.

## 6. Reactions in the Absence of Solvent

In many publications, the hydroxymethylenebisphosphonic acid derivatives were prepared in the absence of any solvent. These reactions are more heterogeneous, and in most cases they cannot be stirred, due to the (almost) solid consistency. Although the yields reported are not wrong, the purities were provided only in a few instances. For this, the conclusions from the yields may be misleading. It can be seen that beside phosphorus trichloride or phosphoryl chloride, there was need for another reactant as well, for example for phosphorous acid or Ms_2_O.

Reacting β-alanine with an unnecessarily large amount of phosphorus trichloride (5 equivalents) and phosphorous acid (3 equivalents) under MW conditions, sodium pamidronate was isolated in a yield of 67% (Table 15/Entry 1) [51]. A rather similar yield (61%) was reported applying the above P-reagents in a 2:1.5 equivalents quantity (Table 15/Entry 2) [52]. Keglevich et al. obtained pure pamidronic acid in a yield of 44%, when both phosphorus trichloride and phosphorous acid were used in a quantity of 2 equivalents [53]. According to our experiences, in most cases it is not possible to prepare the hydroxymethylenebisphosphonic acid derivatives in the absence of solvents.

Alendronic acid derivatives were prepared starting from GABA, *N*-phthalimido-GABA, or *N*-phthalimido-GABA-chloride, but the purities were not reported at all. Using GABA as the starting material, and phosphorus trichloride along with Ms_2_O or phosphorous acid as the other reagent in a molar ratio of 3:3 (PCl_3_:Ms_2_O) and 5:3 (PCl_3_:H_3_PO_3_ under MW conditions), or 2:1.5 (PCl_3_:H_3_PO_3_), the yields were 78%, 78% and 59%, respectively (Table 16/Entries 1–3) [48,51,52]. Starting from *N*-phthalimido-GABA and applying phosphorus trichloride along with phosphorous acid, or this combined with phosphoric acid, crude alendronic acid was obtained in yields of 38% and 57% (Table 16/Entries 4–5) [54]. In one instance, *N*-phthalimido-GABA-chloride was reacted with 2 equivalents of phosphorous acid alone, leading to the target dronic acid in a poor yield of 19% (Table 16/Entry 6) [54]. According to our earlier results [16,21,29,53], phosphorous acid alone is completely unreactive in the synthesis of hydroxymethylenebisphosphonic acid derivatives. Therefore, even the low yield (19%) reported is questionable (Table 16/Entry 6) [54].

The application of 2.9 equivalents of phosphorus trichloride and 1.5 equivalents of phosphorous acid under somewhat different conditions (at 60–65 °C for 1.5 h or at 70–75 °C for 20 h) led to ibandronate in yields of 89% and 82% (Table 17/Entries 1 and 2) [55,56]. A similar result (82%) was reported using a larger excess of the P-reagents (4.1 equivalents of phosphorus trichloride and 2.5 equivalents of phosphorous acid). However, no criterions of purity were provided (Table 17/Entry 3) [57]. Measuring in, even more reagents (5 equivalents of phosphorus trichloride and 3 equivalents of phosphorous acid), and performing the reaction under MW conditions, a somewhat lower yield (72%) was obtained (Table 17/Entry 4) [51]. With a not defined quantity of phosphorus trichloride and 1.3 equivalents of phosphorous acid, sodium ibandronate was prepared in a yield of 68% (Table 17/Entry 5) [58]. The lowest yield of 59% was observed using 3.2 equivalents of phosphoryl chloride and a large excess (9.7 equivalents) of phosphorous acid (Table 17/Entry 6) [59]. The use of this large excess is completely needless, even makes the purification more difficult.

Risedronic acid derivatives were synthesized from PAA. Using phosphorus trichloride and phosphorous acid in a molar ratio of 5:3, the yield was 86% (Table 18/Entry 1) [51]. In one case, morpholine was also added to the reaction mixture beside the P-reagents, and the crude risedronic acid was prepared in a yield of 78% (Table 18/Entry 2) [60]. Yields around 66% were claimed using ca. 2 equivalents of both phosphorus trichloride and phosphorous acid at a temperature of 70–100 °C (Table 18/Entries 3 and 4) [52,61]. Carrying out the reaction applying 3.2 equivalents of phosphoryl chloride and a large excess (9.7 equivalents) of phosphorous acid, the yield was 60% (Table 18/Entry 5) [59].

Reacting IAA, phosphorus trichloride and phosphorous acid in a molar ratio of 1:5:3 under MW conditions, or in a ratio of 1:3:5, zoledronate and zoledronic acid were obtained in a yield of 80% and 61%, respectively. The first yield was related on crude product (Table 19/Entries 1–2) [51,62]. Using 3 equivalents of phosphoryl chloride and 5 equivalents of phosphorous acid, the yield was 79% for the crude, but pure zoledronic acid (Table 19/Entry 3) [62]. The application of phosphoryl chloride and phosphorous acid measured in an excessive quantity (reported also for ibandronic and risedronic acid) led to a yield of 62% (Table 19/Entry 4) [59].

## 7. Methods in ILs

**Scheme 13:**
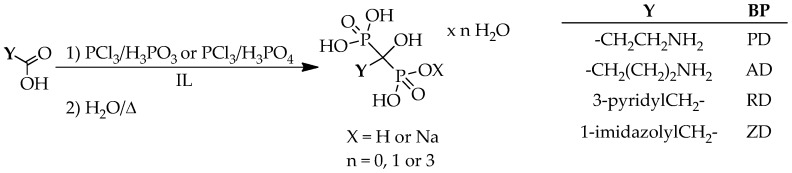
General scheme for the preparation of dronic acid derivatives in ILs.

The ILs are considered green solvents, because of their low vapor pressure, high thermal stability, and, as they can be recycled and reused. Although their use in organic syntheses is spreading, hydroxymethylenebisphosphonic acid derivatives were prepared in ILs only in few cases (Table 20) (Scheme 13).

De Ferra and co-workers investigated the preparation of pamidronic and alendronic acid derivatives (in two cases, the form of the products was not provided), as well as risedronic and zoledronic acid in tributylmethylammonium chloride ([Bu_3_NH][Cl]). Reacting the mixture of 2 equivalents of phosphorus trichloride and 1 equivalent of phosphorous acid with the corresponding carboxylic acid, low yields of the respective dronic acid derivatives were reported (12%–31%) (Table 20/Entries 2 and 4–6) [63]. The Keglevich group reached better results, when synthesizing pamidronic acid in the presence of 1-butyl-3-methylimidazolium hexafluorophosphate ([bmim][PF_6_]). The IL was not used a solvent, only as an additive. Reacting β-alanine with 2:2 or 3:2 equivalents of phosphorus trichloride and phosphorous acid, the optimum amount of the IL was found to be around 0.3–0.6 equivalents. Under such conditions, record yields of 70%–72% were reached (Table 20/Entry 1) [53]. GABA was reacted with 2 equivalents of phosphorus trichloride and 1.5 equivalents of phosphorous acid, in the presence of 0.5 equivalents of various ILs at 60 °C for about 6 h. Depending on the ILs used, the yield of pure alendronate was 92%–94% (Table 20/Entry 3) [64]. The same research group also prepared zoledronic acid in different ILs. For crude zoledronic acid, yields of 90%–92% were reported. In the basic syntheses, IAA was reacted with 2.5 equivalents of phosphorus trichloride and 1.7 equivalents of phosphoric acid in 1.6 equivalents of ILs (Table 20/Entry 7). Carrying out the reaction with the same amount of P-reactants but with [bmim][BF_4_], the yield was 60% for sodium zoledronate (Table 20/Entry 8) [65].

## 8. Processes for the Preparation of Hydroxymethylenebisphosphonic Acid Derivatives in Other Solvents

**Scheme 14:**
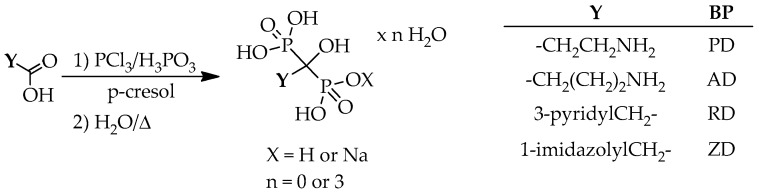
General scheme for the preparation of dronic acid derivatives in *p*-cresol.

In a few instances, the synthesis of hydroxymethylenebisphosphonic acid derivatives was carried out in other solvents, from among *p*-cresol, acetonitrile and *n*-octane should be mentioned. In these cases again the corresponding carboxylic acids were reacted with phosphorus trichloride and phosphorous acid. In the first series let us see the examples carried out in *p*-cresol (Scheme 14).

Applying β-alanine or IAA together with phosphorus trichloride and phosphorous acid in a molar ratio of 3.5:3 and using *p*-cresol as the solvent, crude pamidronic acid was obtained in a yield of 57%, while crude zoledronic acid in a yield of 80% (Table 21/Entries 1 and 5) [66]. Carrying out the syntheses from GABA, and using phosphorus trichloride and phosphorous acid in a ratio of 3.2:1.5 or 3.5:2, alendronate was obtained in a yield of ca. 40% (Table 21/Entries 2 and 3) [67,68]. When PAA was reacted with the mixture of 3.4 equivalents of phosphorus trichloride and 3 equivalents of phosphorous acid, a yield of 59% was reported for risedronic acid (Table 21/Entry 4) [68].

**Scheme 15:**
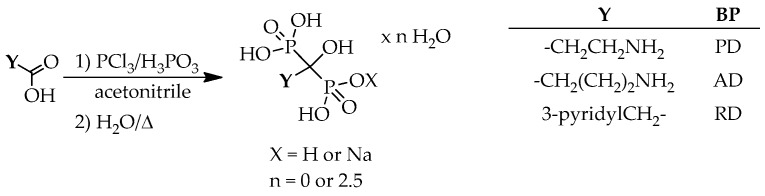
General scheme for the preparation of dronic acid derivatives in acetonitrile.

From among the basic hydroxymethylenebisphosphonates, only pamidronate, alendronate and risedronate was synthesized in acetonitrile (Scheme 15).

In the synthesis of pamidronate, β-alanine and the usual P-reactants were used in a ratio of 1:2:1.5, and the crude product was obtained in a yield of 60% (Table 22/Entry 1) [69]. Applying both phosphorus trichloride and phosphorous acid in amount of 2 equivalents in reaction with GABA, crude alendronate was obtained in a yield of 70% (Table 22/Entry 2) [69]. Risedronic acid was synthesized in a yield of 85% from PAA using phosphorus trichloride and phosphorous acid in a ratio of 2.7:2.4 (Table 22/Entry 3) [38]. When the mixture of 2.7 equivalents of phosphorus trichloride and 2.4 equivalents of phosphorous acid, or 2 equivalents of phosphorus trichloride and 1.2 equivalent of phosphorous acid was reacted with PAA, yields of 77% and 45% were reported for sodium risedronate, respectively (Table 22/Entries 4 and 5) [38,69].

**Scheme 16:**
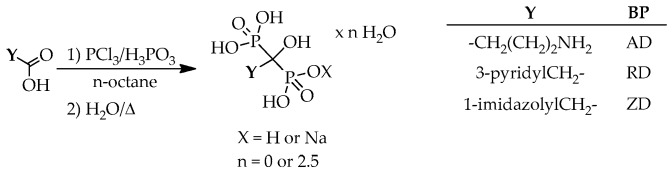
General scheme for the preparation of dronic acid derivatives in *n*-octane.

In a few instances, *n-*octane was also described as the solvent (Scheme 16).

Starting from GABA and PAA, and applying phosphorus trichloride and phosphorous acid in a quantity of ca. 3.5 equivalents, yields of 43% and 96% were reported for alendronic acid and risedronic acid, respectively (Table 23/Entries 1 and 2) [70]. In another combination, the use of phosphoryl chloride and phosphorous acid in a ratio of 3:5 led the risedronate in a yield of 64% (Table 23/Entry 3) [71]. Starting from IAA, and measuring in both phosphorus trichloride and phosphorous acid in a 4.6 molar equivalent quantity, zoledronic acid was obtained in a yield of 65% (Table 23/Entry 4) [70].

The syntheses of the hydroxymethylenebisphosphonates discussed were studied only sporadically in other solvents. For the sake of completeness, we list the special cases in Table 24.

## 9. Conclusions

The methods for the preparation of the most important dronic acid derivatives starting from the corresponding substituted acetic acids and P-reagents, especially phosphorus trichloride and phosphorous acid were surveyed according to the solvents applied. Methanesulfonic acid is a special solvent, as it may enter into reaction also with phosphorus trichloride. The formation of Cl_2_P-OSO_2_Me may mean some kind of activation, and hence there is no need to use phosphorous acid. However, in other cases (typically with sulfolane as the solvent), phosphorus trichloride should be applied together with phosphorous acid to provide more reactive species formed by condensation. The optimum molar ratios of the P-reactants were pointed out. In general, it was a problem during the evaluation of the literature cases that not much stress was placed on purity criterions.

## Figures and Tables

**Table 1:** The dronic acid derivatives discussed in this review.

General Formula	Y	Commercial Name	Abbreviation
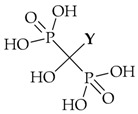	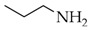	pamidronic acid	**PD**
	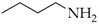	alendronic acid	**AD**
	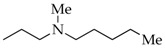	ibandronic acid	**ID**
	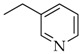	risedronic acid	**RD**
	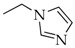	zoledronic acid	**ZD**

**Table 2:** Synthesis of pamidronate from β-alanine in MSA.

Entry	Reagent (Equiv.)	T (°C)	t (h)	Product Form	Purity (%)	Yield (%)	Ref.
1	PCl_3_:H_3_PO_4_ (2:1)	65	16	Na	-	55 ^1^	[13]
2	PCl_3_:H_3_PO_4_ (2:1)	65	18	Na	-	22 ^1^	[14]
3	PCl_3_:H_3_PO_3_ (2.1:1)	65	16–20	Na	-	57 ^1^	[15]
4	PCl_3_ (3.2)	75	24	Na·2H_2_O	99 ^2^	57	[16]

^1^ For the crude product; ^2^ On the basis of acid-base titration.

**Table 3:** Synthesis of alendronic acid derivatives in MSA.

Entry	Starting Material	Reagent (Equiv.)	T (°C)	t (h)	Product form	Purity (%)	Yield (%)	Ref.
1	GABA	PCl_3_:H_3_PO_3_ (2:1)	65	18	Na	-	91 ^1^	[19]
2	GABA	PCl_3_:H_3_PO_3_ (2.4:1.5)	65	20	Na·3H_2_O	99.7 ^2^	90 ^1^	[23]
3	GABA	PCl_3_:H_3_PO_3_ (2.1:1)	65	16–20	Na·3H_2_O	99.7 ^2^	89 ^1^	[15]
4	GABA	PCl_3_:H_3_PO_4_ (2:1)	65	18	acid	-	85 ^1^	[14]
5	GABA	PCl_3_:H_3_PO_3_ (2:1)	65	18	-	-	82 ^1^	[18]
6	GABA	PCl_3_:H_3_PO_3_ (2.1:1)	65	18	-	-	82 ^1^	[20]
7	GABA	PCl_3_ (3.2)	75	12	Na·3H_2_O	93 ^2^	58	[21]
8	GABA	POCl_3_:H_3_PO_3_ (2:3 or 3:3 )	75	12	Na·3H_2_O	97 ^2^	60	[22]
9	GABA	P_2_O_5_:H_3_PO_3_ (1:2)	70	24	Na·3H_2_O	>99.9 ^3^	48	[17]
10	2-Pyrrolidone	PCl_3_ (3.4)	100–105	6	Na·3H_2_O	-	81 ^1^	[24]

^1^ For the crude product; ^2^ On the basis of acid-base or complexometric titration; ^3^ On the basis of HPLC.

**Table 4:** Synthesis of ibandronate from MPA in MSA.

Entry	Reagent (Equiv.)	T (°C)	t (h)	Product Form	Purity (%)	Yield (%)	Ref.
1	PCl_3_:H_3_PO_3_ (5:2)	65–67	24	Na·H_2_O	99.6 ^1^	86	[25]
2	PCl_3_:H_3_PO_3_^2^ (2.5:2)	96	3	Na·H_2_O	99.8 ^1^	60	[26]
3	PCl_3_ (3.2)	75	12	Na	99 ^3^	46	[21]

^1^ On the basis of HPLC; ^2^ in the mixture of MSA and PhCl; ^3^ On the basis of acid-base titration.

**Table 5:** Synthesis of risedronic acid derivatives from PAA in MSA.

Entry	Reagent (Equiv.)	T (°C)	t (h)	Product Form	Purity (%)	Yield (%)	Ref.
1	PCl_3_:H_3_PO_3_^1^ (3:3)	70–72	5	Na·2.5H_2_O	99.9 ^2^	73	[27]
2	PCl_3_:H_3_PO_3_ (2.1:1)	65	16–20	acid	-	38 ^3^	[15]
3	POCl_3_:H_3_PO_3_ (2:3)	75	12	acid	98 ^4^	55	[22]
4	P_2_O_5_:H_3_PO_3_ (1:2)	80–100	72	acid·H_2_O	99.9 ^2^	54	[17]
5	PCl_3_ (3.1)	75	12	acid	92 ^4^ (100 ^2^)	74 ^3^	[28,29]

^1^ in the mixture of MSA and diethyl carbonate; ^2^ On the basis of HPLC; ^3^ For the crude product; ^4^ On the basis of acid-base titration.

**Table 6:** Synthesis of zoledronic acid from IAA in MSA.

Entry	Reagent (Equiv.)	T (°C)	t (h)	Product Form	Purity (%)	Yield (%)	Ref.
1	PCl_3_:H_3_PO_3_ (2.1:1)	65	16–20	acid	-	31 ^1^	[15]
2	PCl_3_ (3.1)	80	3	acid	99 ^2^	53	[29]
3	PCl_3_ (3.2)	80	3	acid	98 ^2^	46	[28]

^1^ For the crude product; ^2^ On the basis of acid-base titration.

**Table 7:** Synthesis of pamidronic acid and alendronic acid in chlorobenzene.

Entry	BP	Starting Material	Reagent (Equiv.)	T (°C)	t (h)	Product Form	Purity (%)	Yield (%)	Ref.
1	PD	β-alanine	PCl_3_:H_3_PO_3_ (1.5:1.5)	100	3	acid	-	59 ^1^	[30]
2	PD	β-alanine	PCl_3_:H_3_PO_4_ (2.4:1.5)	105–110	2	acid	-	53	[33]
3	PD	β-alanine	PCl_3_:H_3_PO_3_ (1.5:1.5)	100	3	acid	-	51	[31]
4	AD	GABA	PCl_3_:H_3_PO_3_ (1.5:1.5)	100	3	acid	-	46	[31]
5	AD	GABA	PCl_3_:H_3_PO_3_ (1.5:1.5)	100	3	acid	-	45	[32]
6	AD	GABA	PCl_3_:H_3_PO_4_ (2:3)	100–110	3	acid	-	18 ^1^	[34]

^1^ For the crude product.

**Table 8:** Synthesis of ibandronic acid derivatives from MPA in chlorobenzene.

Entry	Reagent (Equiv.)	T (°C)	t (h)	Product Form	Purity (%)	Yield (%)	Ref.
1	PCl_3_:H_3_PO_3_ (1.5:1.5)	80	4	Na·H_2_O	-	60	[35]
2	PCl_3_:H_3_PO_3_^1^ (2.5:2)	96	3	Na·H_2_O	99.8 ^2^	60	[26]
3	PCl_3_:H_3_PO_4_ (3:3)	110	11	Na	-	37 ^3^	[36]
4	PCl_3_:H_3_PO_4_ (2:3)	100–110	3	acid	-	6 ^1^	[34]
5	PCl_3_^4^:H_3_PO_3_	85–90	4–5	acid	95 ^2^	3	[26]

^1^ in the mixture of MSA and PhCl; ^2^ On the basis of HPLC; ^3^ For the crude product; ^4^ Two and a half equivalents.

**Table 9:** Synthesis of risedronic acid derivatives from PAA in chlorobenzene.

Entry	Reagent (Equiv.)	T (°C)	t (h)	Product Form	Purity (%)	Yield (%)	Ref.
1	PCl_3_:H_3_PO_3_ (3.5:3.5)	90–95	2.5	acid	-	96 ^1^	[37]
2	PCl_3_:H_3_PO_3_ (1.5:1.5)	80	4	acid	-	68 ^1^	[35]
3	PCl_3_:H_3_PO_3_ (2.4:2.4)	85–90	-	acid	84 ^2^	58 ^1^	[38]
4	PCl_3_:H_3_PO_3_ (3:3.5)	-	2.5	Na·2.5H_2_O	-	52	[39]
5	PCl_3_^3^:H_3_PO_3_	100	6	acid	-	52 ^1^	[40]

^1^ For the crude product; ^2^ On the basis of HPLC; ^3^ Two equivalents.

**Table 10:** Synthesis of zoledronic acid from IAA in chlorobenzene.

Entry	Reagent (Equiv.)	T (°C)	t (h)	Product Form	Purity (%)	Yield (%)	Ref.
1	PCl_3_:H_3_PO_3_ (3.7:3.7)	100	1	acid	-	100 ^1^	[41]
2	PCl_3_:H_3_PO_4_ (4.7:2.9)	80	2–2.5	acid	-	79 ^1^	[42]
3	PCl_3_:H_3_PO_4_ (2:3)	100–110	3	acid	-	67 ^1^	[34]
4	PCl_3_:H_3_PO_4_ (3:1.9)	100	3	acid	-	41	[43]

^1^ For the crude product.

**Table 11:** Synthesis of pamidronic and alendronic acid derivatives in sulfolane.

Entry	BP	Starting Material	Reagent (Equiv.)	T (°C)	t (h)	Product Form	Purity (%)	Yield (%)	Ref.
1	PD	β-alanine	PCl_3_:H_3_PO_3_ (3:3)	65	3.5	-	-	72 ^1^	[44]
2	PD	β-alanine	PCl_3_:H_3_PO_3_ (3:3)	65 ^2^	0.05	-	-	64 ^1^	[44]
3	PD	β-alanine	PCl_3_:H_3_PO_3_ (2:2)	75	12	acid	100 ^3^	63	[16]
4	PD	β-alanine	PCl_3_:H_3_PO_3_ (3.4:1.5)	63–67	3	2Na·5H_2_O	>99	48	[45]
5	AD	GABA	PCl_3_:H_3_PO_3_ (3.4:1.5)	63–67	3	Na·3H_2_O	>99	69 ^1^	[45]
6	AD	GABA	PCl_3_:H_3_PO_3_ (2.5:3.5)	60–65	12	Na·3H_2_O	>99 ^4^	55	[46]
7	AD	GABA	PCl_3_:H_3_PO_3_ (3:3)	65 ^2^	0.1	-	-	41 ^1^	[44]
8	AD	GABA	PCl_3_:H_3_PO_3_ (3:3)	65	3.5	-	-	38 ^1^	[44]

^1^ For the crude product; ^2^ MW heating; ^3^ On the basis of acid-base titration; ^4^ On the basis of HPLC.

**Table 12:** Synthesis of risedronic and zoledronic acid derivatives in sulfolane.

Entry	BP	Starting Material	Reagent (Equiv.)	T (°C)	t (h)	Product Form	Purity (%)	Yield (%)	Ref.
1	RD	PAA	PCl_3_:H_3_PO_3_ (3:3)	65 ^1^	0.05	acid·H_2_O	-	74 ^2^	[44]
2	RD	PAA	PCl_3_:H_3_PO_3_ (3.4:1.5)	63–67	3	Na	>99	54	[45]
3	ZD	IAA	PCl_3_:H_3_PO_3_ (3:3)	65 ^1^	0.05	-	-	70 ^2^	[44]
4	ZD	IAA	PCl_3_:H_3_PO_3_ (3:3)	65	3.5	-	-	67 ^2^	[44]
5	ZD	IAA	PCl_3_:H_3_PO_3_ (3.4:1.5)	63–67	3	acid·H_2_O	>99	71 ^2^	[45]

^1^ Under MW irradiation; ^2^ For the crude product.

**Table 13:** Synthesis of alendronic acid derivatives and ibandronate in toluene.

Entry	BP	Starting Material	Reagent (Equiv.)	T (°C)	t (h)	Product Form	Purity (%)	Yield (%)	Ref.
1	AD	GABA	PCl_3_:H_3_PO_3_^1^ (1.6:1)	75	4	acid	-	56 ^2^	[47]
2	AD	GABA	PCl_3_:Ms_2_O (3:3)	70	9.5	Na·3H_2_O	-	66	[48]
3	ID	MPA	PCl_3_:H_3_PO_3_ (3.7:4.2)	80–85	7–8	Na	-	44	[49]

^1^ in the mixture of toluene and PEG 400; ^2^ For the crude product.

**Table 14:** Synthesis of risedronic acid from PAA in toluene

Entry	Reagent (Equiv.)	T (°C)	t (h)	Product Form	Purity (%)	Yield (%)	Ref.
1	H_3_PO_3_:T3P (3.1:2.5)	95	20.5	acid·H_2_O	99.6 ^1^	90 ^2^	[50]
2	H_3_PO_3_:POCl_3_ (3.1:3.1)	95	20.5	acid·H_2_O	98.5 ^1^	81 ^2^	[50]
3	H_3_PO_3_:Ms_2_O (3.1:3)	95	20.5	acid·H_2_O	95.9 ^1^	34 ^2^	[50]
4	H_3_PO_3_:Ms_2_O:POCl_3_ (3.1:2:1)	95	20.5	acid·H_2_O	99.2 ^1^	62 ^2^	[50]
5	H_3_PO_3_:Ms_2_O:PCl_5_ (3.1:1:1)	95	20.5	acid·H_2_O	98.3 ^1^	58 ^2^	[50]
6	PCl_3_ (3.2)	110	6	acid	90.1 ^3^	15 ^2^	[12]

^1^ On the basis of HPLC; ^2^ For the crude product; ^3^ On the basis of acid-base titration.

**Table 15:** Synthesis of pamidronic acid derivatives from β-alanine in neat.

Entry	Reagent (Equiv.)	T (°C)	t (h)	Product Form	Purity (%)	Yield (%)	Ref.
1	PCl_3_:H_3_PO_3_ (5:3)	80 ^1^	0.05	Na	-	67 ^2^	[51]
2	PCl_3_:H_3_PO_3_ (2:1.5)	100	4	acid	-	61 ^2^	[52]
3	PCl_3_:H_3_PO_3_ (2:2)	75	3	acid	>99 ^3^	44	[53]

^1^ Under MW conditions; ^2^ For the crude product; ^3^ On the basis of acid-base titration.

**Table 16:** Synthesis of alendronic acid derivatives in the absence of solvents.

Entry	Starting Material	Reagent (Equiv.)	T (°C)	t (h)	Product Form	Purity (%)	Yield (%)	Ref.
1	GABA	PCl_3_:Ms_2_O (3:3)	70–100	7	Na·3H_2_O	-	78	[48]
2	GABA	PCl_3_:H_3_PO_3_ (5:3)	80 ^1^	0.05	Na	-	78 ^2^	[51]
3	GABA	PCl_3_:H_3_PO_3_ (2:1.5)	100	4	acid	-	59 ^2^	[52]
4	*N*-phthalimido-GABA	PCl_3_:H_3_PO_3_ (2:5)	80	3	acid	-	38 ^2^	[54]
5	*N*-phthalimido-GABA	PCl_3_:H_3_PO_3_:H_3_PO_4_ (2:1.5:4)	80	3	acid	-	57 ^2^	[54]
6	*N*-phthalimido-GABA-chloride	H_3_PO_3_ (2)	130	4	acid	-	19 ^2^	[54]

^1^ Under MW conditions; ^2^ For the crude product.

**Table 17:** Synthesis of ibandronic acid derivatives from MPA in the absence of any solvent.

Entry	Reagent (Equiv.)	T (°C)	t (h)	Product Form	Purity (%)	Yield (%)	Ref.
1	PCl_3_:H_3_PO_3_ (2.9:1.5)	60–65	1.5	Na	-	89	[55]
2	PCl_3_:H_3_PO_3_ (2.9:1.5)	70–75	20	Na·H_2_O	-	82	[56]
3	PCl_3_:H_3_PO_3_ (4.1:2.5)	75	5	Na	-	82 ^1^	[57]
4	PCl_3_:H_3_PO_3_ (5:3)	80 ^2^	0.05	Na	-	72 ^1^	[51]
5	PCl_3_:H_3_PO_3_^3^	75	6	Na·H_2_O	-	68 ^1^	[58]
6	POCl_3_:H_3_PO_3_ (3.2:9.7)	60–70	24	acid	-	59 ^1^	[59]

^1^ For the crude product; ^2^ Under MW conditions; ^3^ 1.3 equivalents of phosphorous acid were used.

**Table 18:** Synthesis of risedronic acid derivatives from PAA in the absence of solvent.

Entry	Reagent (Equiv.)	T (°C)	t (h)	Product Form	Purity (%)	Yield (%)	Ref.
1	PCl_3_:H_3_PO_3_ (5:3)	80 ^1^	0.05	Na	-	86 ^2^	[51]
2	PCl_3_:H_3_PO_3_:morpholine (2:5:1)	68–75	2.5–4	acid	-	78 ^2^	[60]
3	PCl_3_:H_3_PO_3_ (2:1.5)	100	4	acid	-	67 ^2^	[52]
4	PCl_3_:H_3_PO_3_ (2.5:2.2)	70–75	8	Na·2.5H_2_O	99.9 ^3^	65	[61]
5	POCl_3_:H_3_PO_3_ (3.2:9.7)	60–70	24	acid	-	60 ^2^	[59]

^1^ Under MW conditions; ^2^ For the crude product; ^3^ On the basis of HPLC.

**Table 19:** Synthesis of zoledronic acid derivatives from IAA under solvent-free conditions.

Entry	Reagent (Equiv.)	T (°C)	t (h)	Product Form	Purity (%)	Yield (%)	Ref.
1	PCl_3_:H_3_PO_3_ (5:3)	80 ^1^	0.05	Na	-	80 ^2^	[51]
2	PCl_3_:H_3_PO_3_ (3:5)	75	6	acid	99.9 ^3^	61	[62]
3	POCl_3_:H_3_PO_3_ (3:5)	75	5	acid	99.9 ^3^	79 ^2^	[62]
4	POCl_3_:H_3_PO_3_ (3.2:9.7)	60–70	24	acid	-	62 ^2^	[59]

^1^ Under MW conditions; ^2^ For the crude product; ^3^ On the basis of HPLC.

**Table 20:** Synthesis of hydroxymethylenebisphosphonic acid derivatives in the presence of ILs.

Entry	BP	Ils (Equiv.)	Starting Material	Reagent (Equiv.)	T (°C)	t (h)	Product Form	Purity (%)	Yield (%)	Ref.
1	PD	[bmim][PF_6_] (0.3 or 0.6)	β-alanine	PCl_3_:H_3_PO_3_ (2:2 or 3:2)	75	3	acid	>99 ^1^	70–72	[53]
2	PD	[Bu_3_NH][Cl]	β-alanine	PCl_3_:H_3_PO_3_ (2:1)	60	2	-	-	26	[63]
3	AD	various Ils ^2^ (0.5)	GABA	PCl_3_:H_3_PO_3_ (2:1.5)	60	6	Na·3H_2_O	>99 ^3^	92–94	[64]
4	AD	[Bu_3_NH][Cl]	GABA	PCl_3_:H_3_PO_3_ (2:1)	60	2	-	-	31	[63]
5	RD	[Bu_3_NH][Cl]	PAA	PCl_3_:H_3_PO_3_ (2:1)	80	2	acid	-	12	[63]
6	ZD	[Bu_3_NH][Cl]	IAA	PCl_3_:H_3_PO_3_ (2:1)	80	2	acid	-	26	[63]
7	ZD	various Ils ^4^ (1.6)	IAA	PCl_3_:H_3_PO_4_ (2.5:1.7)	65	4	acid	-	90–92 ^5^	[65]
8	ZD	[bmim][BF_4_] (1.6)	IAA	PCl_3_:H_3_PO_4_ (2.5:1.7)	65	4	Na·H_2_O	99.8 ^3^	60	[65]

^1^ On the basis of acid-base titration; ^2^ [bmim][BF_4_], [bmim][PF_6_], 1-hydroxyethyl-2,3-dimethyl-imidazolium chloride (LOH), 1-propyl-3-carbonitrile-imidazolium chloride (LCN), 1-carboxyethyl-3-methyl-imidazolium chloride (LOOH); ^3^ On the basis of HPLC; ^4^ *N*-ethylpyridine tetrafluoroborate [EPy][BF_4_], [bmim][PF_6_], LOH, LCN, LOOH; ^5^ For the crude product.

**Table 21:** Synthesis of hydroxymethylenebisphosphonic acid derivatives in *p*-cresol.

Entry	BP	Starting Material	Reagent (Equiv.)	T (°C)	t (h)	Product Form	Purity (%)	Yield (%)	Ref.
1	PD	β-alanine	PCl_3_:H_3_PO_3_ (3.5:3)	65–70	5	acid	-	57 ^1^	[66]
2	AD	GABA	PCl_3_:H_3_PO_3_ (3.2:1.5)	75	5	Na·3H_2_O	99.9 ^2^	42	[67]
3	AD	GABA	PCl_3_:H_3_PO_3_ (3.5:2)	50–55	2	Na·3H_2_O	99 ^2^	39	[68]
4	RD	PAA	PCl_3_:H_3_PO_3_ (3.4:3)	65–70	4	acid	-	59 ^1^	[68]
5	ZD	IAA	PCl_3_:H_3_PO_3_ (3.5:3)	65–70	5	acid	-	80 ^1^	[66]

^1^ For the crude product; ^2^ On the basis of HPLC.

**Table 22:** Synthesis of hydroxymethylenebisphosphonates in acetonitrile.

Entry	BP	Starting Material	Reagent (Equiv.)	T (°C)	t (h)	Product Form	Purity (%)	Yield (%)	Ref.
1	PD	β-alanine	PCl_3_:H_3_PO_3_ (2:1.5)	70–75	6–9	Na	-	60 ^1^	[69]
2	AD	GABA	PCl_3_:H_3_PO_3_ (2:2)	70–75	6–9	Na	-	70 ^1^	[69]
3	RD	PAA	PCl_3_:H_3_PO_3_ (2.7:2.4)	82	5	acid	98.6 ^2^	85 ^1^	[38]
4	RD	PAA	PCl_3_:H_3_PO_3_ (2.7:2.4)	82	5	Na	99.3 ^2^	77 ^1^	[38]
5	RD	PAA	PCl_3_:H_3_PO_3_ (2:1.2)	70–75	6–9	Na·2.5H_2_O	99.7 ^2^	45	[69]

^1^ For the crude product; ^2^ On the basis of HPLC.

**Table 23:** Synthesis of hydroxymethylenebisphosphonic acid derivatives in *n-*octane.

Entry	BP	Starting Material	Reagent (Equiv.)	T (°C)	t (h)	Product Form	Purity (%)	Yield (%)	Ref.
1	AD	GABA	PCl_3_:H_3_PO_3_ (3.6:3.5)	90–95	-	acid	-	43 ^1^	[70]
2	RD	PAA	PCl_3_:H_3_PO_3_ (3.5:3.5)	90–95	-	acid	-	96 ^1^	[70]
3	RD	PAA	POCl_3_:H_3_PO_3_ (3:5)	85–90	3–4	Na·2.5H_2_O	99.9 ^2^	64	[71]
4	ZD	IAA	PCl_3_:H_3_PO_3_ (4.6:4.6)	90–95	-	acid	-	65	[70]

^1^ For the crude product; ^2^ On the basis of HPLC.

**Table 24:** Synthesis of hydroxymethylenebisphosphonic acid derivatives in other solvents.

Solvent	PD	AD	ID	RD	ZD
benzenesulfonic acid	[72]	[72]			
trifluoromethanesulfonic acid		[67]			
4-chlorotoluene			[26]		
ethyl acetate				[50]	
xylene	[73]				
cyclohexane			[26]		[42]
methylcyclohexane				[74]	
decalin				[74]	
tetralin				[74]	
anisole		[75,76]	[26]		
dimethylformamide	[77]				
dioxane		[77]		[70]	[70]
diphenyl ether		[78]		[78]	[78]
poly(ethylene glycol) methyl ether 350		[47]			
tetrahydrofuran		[79]			
phenol		[66,68]		[68]	
4-nitrophenol		[68]		[66,68]	
2,6-di-terc-butil-4-metilfenol (BHT)		[68]			
nonylphenol ethoxylate				[80]	
triethyl phosphate		[67]			
tricresyl phosphate		[67]			
diethyl carbonate			[81,82,83]	[27]	
propylene carbonate					[84]
in the mixture of propylene carbonate and PEG 600					[84]
PEG 400					[41]
dimethoxymethane					[41,85]
diethoxymethane			[86]		
diglyme					[85]
1,2-dichloroethane					[42]
*N*,*N*′-dimethylurea				[87]	[87]
sunflower oil			[88,89]	[80]	
olive oil				[80]	
in the presence of paraffin				[80]	
silicon oil			[90]		[41]
